# Combining Observations and Models: A Review of the CARDAMOM Framework for Data‐Constrained Terrestrial Ecosystem Modeling

**DOI:** 10.1111/gcb.70462

**Published:** 2025-08-26

**Authors:** Matthew A. Worden, T. Eren Bilir, A. Anthony Bloom, Jianing Fang, Lily P. Klinek, Alexandra G. Konings, Paul A. Levine, David T. Milodowski, Gregory R. Quetin, T. Luke Smallman, Yinon M. Bar‐On, Renato K. Braghiere, Cédric H. David, Nina A. Fischer, Pierre Gentine, Tim J. Green, Ayanna Jones, Junjie Liu, Marcos Longo, Shuang Ma, Troy S. Magney, Elias C. Massoud, Vasileios Myrgiotis, Alexander J. Norton, Nick Parazoo, Elahe Tajfar, Anna T. Trugman, Mathew Williams, Sarah Worden, Wenli Zhao, Songyan Zhu

**Affiliations:** ^1^ Department of Earth System Science Stanford University Stanford California USA; ^2^ Jet Propulsion Laboratory California Institute of Technology Pasadena California USA; ^3^ Department of Earth and Environmental Engineering Columbia University New York New York USA; ^4^ Department of Plant Sciences University of California Davis California USA; ^5^ School of Geosciences University of Edinburgh Edinburgh UK; ^6^ Department of Geography University of California Santa Barbara California USA; ^7^ National Centre for Earth Observation University of Edinburgh Edinburgh UK; ^8^ Division of Geological and Planetary Sciences California Institute of Technology Pasadena California USA; ^9^ Department of Earth and Planetary Sciences Weizmann Institute of Science Rehovot Israel; ^10^ School of Mathematics University of Edinburgh Edinburgh UK; ^11^ Department of Atmospheric Sciences Howard University Washington DC USA; ^12^ Climate and Ecosystem Sciences Division Lawrence Berkeley National Laboratory Berkeley California USA; ^13^ Joint Institute for Regional Earth System Science and Engineering University of California Los Angeles Los Angeles USA; ^14^ Department of Forest Management University of Montana Missoula Montana USA; ^15^ Oak Ridge National Laboratory Oak Ridge Tennessee USA; ^16^ UK Centre for Ecology and Hydrology Midlothian UK; ^17^ Research School of Biology Australian National University Canberra Australian Capital Territory Australia; ^18^ School of Geography and Environmental Science University of Southampton Southampton UK

**Keywords:** Bayesian inference, CARDAMOM, DALEC, data assimilation, data‐constrained model, model‐data fusion

## Abstract

The rapid increase in the volume and variety of terrestrial biosphere observations (i.e., remote sensing data and in situ measurements) offers a unique opportunity to derive ecological insights, refine process‐based models, and improve forecasting for decision support. However, despite their potential, ecological observations have primarily been used to benchmark process‐based models, as many past and current models lack the capability to directly integrate observations and their associated uncertainties for parameterization. In contrast, data assimilation frameworks such as the CARbon DAta MOdel fraMework (CARDAMOM) and its suite of process‐based models, known as the Data Assimilation Linked Ecosystem Carbon Model (DALEC), are specifically designed for model‐data fusion. This review, motivated by a recent CARDAMOM community workshop, examines the development and applications of CARDAMOM, with an emphasis on its role in advancing ecosystem process understanding. CARDAMOM employs a Bayesian approach, using a Markov Chain Monte Carlo algorithm to enable data‐driven calibration of DALEC parameters and initial states (i.e., carbon pool sizes) through observation operators. CARDAMOM's unique ability to retrieve localized model process parameters from diverse datasets—ranging from in situ measurements to global satellite observations—makes it a highly flexible tool for analyzing spatially variable ecosystem responses to environmental change. However, assimilating these data also presents challenges, including data quality issues that propagate into model skill, as well as trade‐offs between model complexity, parameter equifinality, and predictive performance. We discuss potential solutions to these challenges, such as reducing parameter equifinality by incorporating new observations. This review also offers community recommendations for incorporating emerging datasets, integrating machine learning techniques, strengthening collaboration with remote sensing, field, and modeling communities, and expanding CARDAMOM's relevance for localized ecosystem monitoring and decision‐making. CARDAMOM enables a deep, mechanistic understanding of terrestrial ecosystem dynamics that cannot be achieved through empirical analyses of observational datasets or weakly constrained models alone.

## Graphical abstract

The rapid increase in the volume and variety of terrestrial biosphere observations offers a unique opportunity to derive ecological insights, refine process‐based models, and improve forecasting for decision support. Data assimilation frameworks such as the CARbon DAta MOdel fraMework (CARDAMOM) are specifically designed to take advantage of this rapid increase in data through model‐data fusion. In this review, we examine the development and applications of CARDAMOM, with an emphasis on its role in advancing ecosystem process understanding, and provide community recommendations for CARDAMOM's development, including, among others, pathways for incorporating emerging datasets and machine learning techniques.

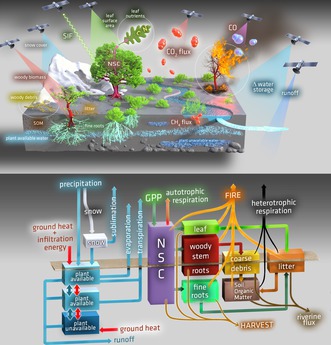

## 1 Introduction

Terrestrial biogeochemical cycles are an integral part of the earth system, regulating the global carbon cycle, buffering fossil fuel emissions, and providing planetary‐scale ecosystem services (Bonan 2008; Friedlingstein et al. 2023, 2024). Yet terrestrial ecosystems—together with human behavior and cloud feedbacks—remain among the most uncertain factors in decadal‐to‐centennial earth system forecasts (Intergovernmental Panel on Climate Change [IPCC] 2023). Over recent decades, fundamental insights into ecosystem processes have arisen from careful observations and theoretical progress, including leaf‐to‐planetary‐scale observation of photosynthetic processes (e.g., Ryu et al. 2019; Baldocchi 2020), atmospheric greenhouse gas inversions (e.g., Peylin et al. 2013; Basu et al. 2020), and the formulation of novel paradigms of soil–plant‐atmosphere biophysical process representations in numerical models (e.g., Bonan 2019). These efforts have produced increasingly complex global vegetation models (Fisher and Koven 2020), but their sophistication often depends on parameterizations that are weakly linked to observations and assumed to be largely invariant in space, limiting model accuracy (Bonan and Doney 2018; Famiglietti et al. 2021). As a result, parameter uncertainty contributes as much to model uncertainty as model structure does (Quetin et al. 2020; Smallman et al. 2021). Consequently, ecosystem observations—and their integration—are crucial for refining and validating models at the planetary scale (Eyring et al. 2019; Schimel and Carroll 2024).

Observations of the terrestrial biosphere have dramatically increased in the last several decades, offering datasets from remote sensing and in situ measurements (e.g., Worden et al. 2021). In situ (i.e., field‐based) observing networks offer numerous overlapping complementary observations, including biomass, leaf area, plant traits, and also high temporal frequency information (hourly to daily), such as soil moisture measurements and energy, carbon, and water exchange estimates from eddy covariance (Pastorello et al. 2020). Compiled databases of in situ plant trait information (Kattge et al. 2020) and spatially continuous maps of interpolated soil and vegetation characteristics (Butler et al. 2017; Poggio et al. 2021) are also available. Satellite‐based observations (e.g., Smallman et al. 2022; Williams 2022) provide the means to monitor the entire land surface with increasing temporal frequency, spatial granularity, and over an ever‐growing time span. Satellite observations can offer insights into the biological and physical states of the land surface with appropriate calibration, thereby complementing in situ observations and enhancing our understanding of ecosystem dynamics (Sellers et al. 2018; Schimel et al. 2019). Despite their value, all observations have limitations in terms of errors, bias, uncertainties, and gaps in space and time, which often remain poorly understood or characterized (Hollinger and Richardson 2005; Loescher et al. 2006; Lasslop et al. 2008; Morrison 2016; Zhao et al. 2020; Araza et al. 2023). Furthermore, in situ measurements are spatially biased toward northern latitudes (Schimel et al. 2015) and have small spatial extents that create representativeness errors when scaling (Chu et al. 2021). These biases can propagate into satellite retrieval algorithms. Finally, satellite observations provide an incomplete picture of terrestrial ecosystem cycling. Observations of different components are typically temporally separate, and many key states (e.g., belowground biomass) and fluxes (e.g., respiration) remain unobservable from space and are challenging to quantify in situ.

Process‐based models of the terrestrial biosphere provide a systemic view of terrestrial ecosystems, bridging the gap between observations in space and time. These models represent theories of ecosystem functioning (e.g., Bonan 2019; Ge et al. 2022) and, when combined with observations, allow us to improve our understanding of the Earth system. They also offer a vital capacity to explore climate, disturbance, and management scenarios to predict likely future responses of ecosystem states and services. These “what‐if” scenarios have informed IPCC reports (IPCC 2023), underpinning international negotiations aimed at tackling climate change and supporting policymaking. However, most process‐based models are weakly constrained by sparse observations from a limited number of sites, primarily using observational data for benchmarking and initializing conditions (e.g., Collier et al. 2018; Seiler et al. 2022; Braghiere et al. 2022). As a result, they often have weakly constrained parameters and lack uncertainty estimates (Hourdin et al. 2017; Bonan and Doney 2018; Famiglietti et al. 2021). Moreover, without adequate calibration, rigorous evaluation of competing hypotheses embedded in process‐based models becomes impossible (e.g., Fisher and Koven 2020; Famiglietti et al. 2021; Smallman et al. 2021; Norton et al. 2023).

Multiple studies have hypothesized that the inability to effectively integrate observations with current models underpins much of the divergence in carbon cycling forecasts (e.g., Lovenduski and Bonan 2017; Lovenduski et al. 2019; Arora et al. 2020; Bonan et al. 2021; Bonan et al. 2024; Smallman et al. 2021). This hypothesis is a key motivation for advancing model‐data fusion approaches, which can enhance the representation of complex environmental dynamics and reduce uncertainties in ecosystem and climate projections (Schimel and Carroll 2024). Moreover, integrating models with observations allows for better characterization, explanation, and communication of uncertainties in both models and data (e.g., Keenan et al. 2011; Lahoz and Schneider 2014; Yin et al. 2020). The primary challenge lies in integrating observations with models in a manner that effectively informs the underlying processes (Schneider et al. 2017). This challenge has stimulated the development of a number of different model‐data fusion approaches (e.g., Bloom et al. 2016; Scholze et al. 2016; Fox et al. 2018; Luo and Smith 2022). These approaches and their associated tradeoffs are discussed in more detail in Section 3.

Here, we review the CARbon DAta MOdel fraMework (CARDAMOM), a model‐data fusion framework developed over 20 years across multiple institutions that draws on spatially and temporally explicit observations and ecological knowledge to infer ecosystem states, process parameterizations, and biogeochemical dynamics (Bloom and Williams 2015; Bloom et al. 2016). CARDAMOM's unique ability to infer parameters from observations across varied spatial and temporal scales enables a plant functional type–agnostic approach that captures ecosystem functional gradients from localized studies to broader regional and global analyses (e.g., Myrgiotis et al. 2021; Yang et al. 2022; Bloom et al. 2016). First, we describe the CARDAMOM framework and provide examples of its applications, showcasing how the integration of models and observations has been used to derive insights that neither could achieve independently. Second, we highlight CARDAMOM's unique capacities by contrasting them, where appropriate, with those of other terrestrial ecosystem modeling approaches that assimilate data. Third, we discuss the challenges involved in working with the CARDAMOM system. Finally, we explore future opportunities—including machine and/or deep learning techniques, novel satellite observations, and emergent constraints derived from in situ observing networks—to further enhance ecosystem analyses, extract ecological insights, support robust model development, and overcome limitations.

## 2 Overview, Historical Development, and Applications

### 2.1 Framework Overview

**FIGURE 1:**
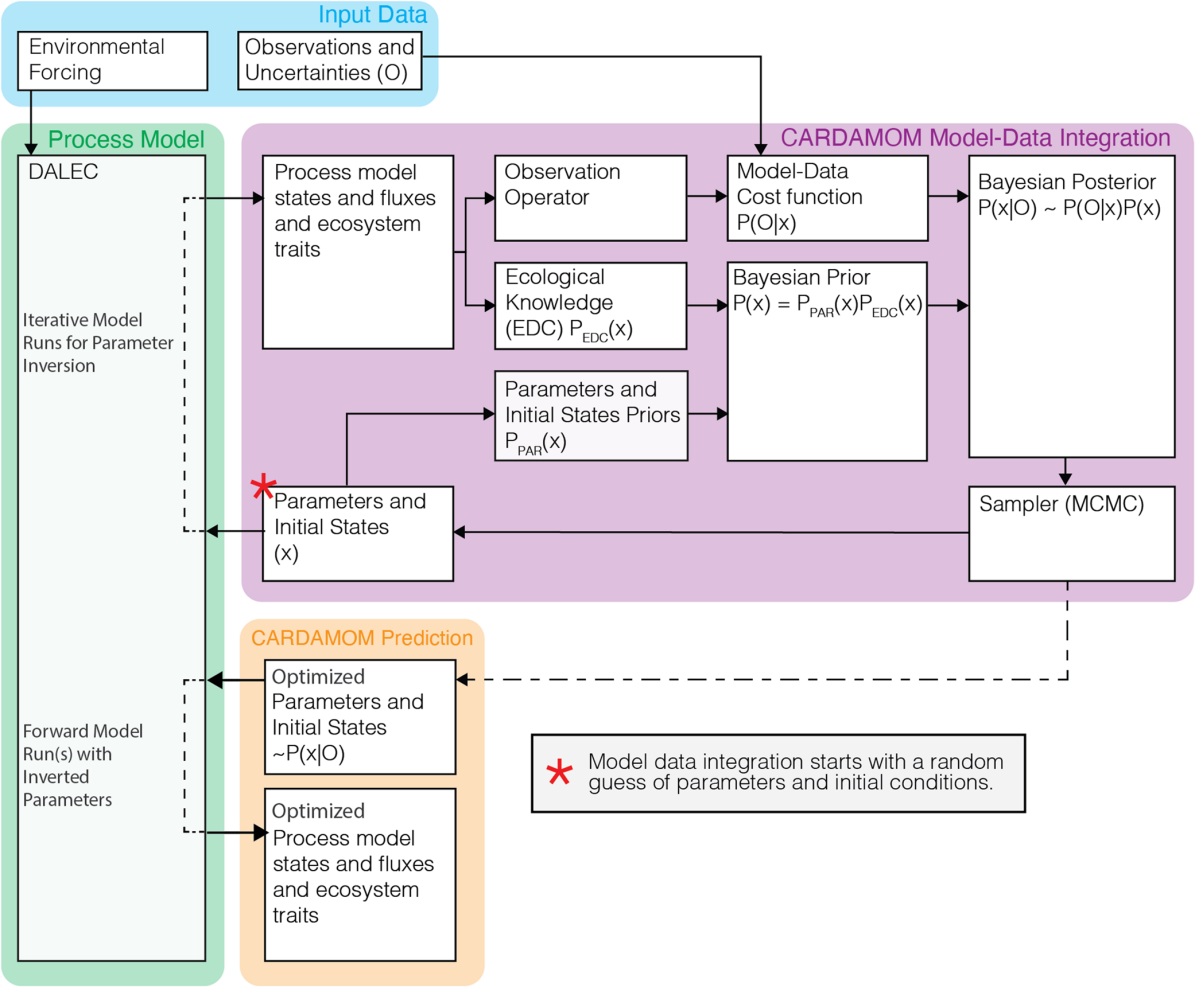
Illustration of the CARbon DAta MOdel FraMework (CARDAMOM), describing its inputs (blue), its process‐based model (green), the parameter and initial state optimization process (purple), and its outputs using optimized parameters (orange).

The CARDAMOM framework consists of three main components: (i) a process‐based model of the terrestrial ecosystem, called the Data Assimilation Linked Ecosystem Carbon Model (DALEC) (Figure 1, green box); (ii) observation operators that link the process‐based model to ecosystem measurements (Figure 1, blue box); and (iii) a Bayesian inference algorithm that integrates observations and their uncertainties, prior knowledge of parameter values or ranges, and prior knowledge of ecological function (referred to as ecological and dynamical constraints; hereafter EDC) to constrain model states and process parameters (Figure 1, purple box; Bloom et al. 2016; Bloom and Williams 2015; Yang et al. 2022). Working with CARDAMOM begins with a model‐data fusion step (Figure 1), during which the three main components iteratively retrieve parameters and initial states that align DALEC with observations and their associated uncertainties. Once CARDAMOM has retrieved ensembles of parameters consistent with the observations, these parameter ensembles can be used by DALEC in a forward run to generate data‐constrained ensembles of states and fluxes (Figure 1, orange box). CARDAMOM is implemented in a modular manner so that the process‐based model, observation operators, and Bayesian inference algorithm can be swapped for alternative approaches and are distinct enough for users to use each part individually.

#### 2.1.1 The Process‐Based Model DALEC as the Backbone of CARDAMOM

CARDAMOM uses a suite of intermediate complexity process‐based models collectively known as DALEC. The DALEC suite represents terrestrial ecosystems with a pool‐based mass balance approach, where carbon, water, and energy states evolve through parameterized ordinary differential equations (Williams 2022). Its relatively simple structure makes it straightforward to develop and implement new theoretical approaches as new observations become available to test competing hypotheses (e.g., Famiglietti et al. 2021). Currently, more than a dozen published model versions exist, each featuring a different level of complexity and reflecting various theoretical assumptions about ecosystem functioning. These versions include different representations of photosynthesis (Quetin, Famiglietti, et al. 2023; Williams et al. 2005; Smallman and Williams 2019), hydrological processes (Spadavecchia et al. 2011; Bloom et al. 2016; Smallman and Williams 2019; Yang et al. 2022), respiration processes (Rowland et al. 2014; Quetin, Famiglietti, et al. 2023; Yin et al. 2020; Ma et al. 2023), and phenological processes (Smallman et al. 2017; Exbrayat et al. 2018; Norton et al. 2023; Bloom et al. 2020). Users select the most appropriate model from the DALEC suite and modify it as needed based on their research questions and available data, a process that also allows different model structures to be compared and evaluated against observations (e.g., Famiglietti et al. 2021; Worden et al. 2024). Although the DALEC models are the only process‐based models integrated into CARDAMOM, other models could be incorporated by adapting them to accept input parameters and initial states from CARDAMOM's Markov Chain Monte Carlo (MCMC) sampler and to produce output states and fluxes comparable to observations via CARDAMOM's observation operators (Figure 1).

DALEC is driven by environmental forcing data. Forcing data varies depending on the model version but typically includes meteorological variables and exogenous processes. Examples of meteorological forcings include minimum and maximum air temperatures, incoming short‐wave radiation, atmospheric CO_2_ concentration, vapor pressure deficit, precipitation, and wind speed. Exogenous processes typically encompass disturbance or management activities such as fire (Exbrayat et al. 2018) or biomass extraction from forests (Smallman et al. 2017). Using these forcings, along with the parameters and initial states provided by the MCMC sampler, DALEC generates states and fluxes that can be compared to observations through an observation operator (Figure 1).

#### 2.1.2 Connecting Model States and Fluxes to Varied Observations With Flexible Resolution

CARDAMOM can assimilate any observation type, provided that a suitable observation operator (Figure 1) is developed to link the observation to DALEC states or fluxes. Possible observations include in situ measurements such as net and gross fluxes of gases (CO_2_, water, and methane), biomass, foliage, leaf area index, fine roots, litter, and soil carbon (Smallman et al. 2017; Famiglietti et al. 2021; Yang et al. 2022; George‐Chacon et al. 2023; Worden et al. 2024). CARDAMOM is equally versatile in assimilating satellite‐based observations, including leaf area index, equivalent water thickness, solar‐induced fluorescence, and net biosphere exchange (Bloom et al. 2020). Additionally, CARDAMOM's observation operators can be tailored to specific comparisons, such as linking solar‐induced fluorescence with DALEC gross primary productivity or separating the monthly and interannual variability of net biosphere exchange (Quetin et al. 2020).

The temporal and spatial resolution at which CARDAMOM is applied is flexible. CARDAMOM has been applied at daily (Myrgiotis et al. 2021; Revill et al. 2021), weekly (Rowland et al. 2014; Myrgiotis et al. 2020), and monthly (Bloom et al. 2016; Yang et al. 2022; Au et al. 2023) time steps. Spatial resolution varies widely, from multiple degree‐scale grids (Bloom et al. 2016; Quetin, Famiglietti, et al. 2023) down to hectares and tens of meters (Stettz et al. 2022; Au et al. 2023; Myrgiotis et al. 2022).

CARDAMOM's flexible temporal resolution, observation operators, and optimized parameters and initial states enable it to reproduce ecosystem responses across multiple timescales. The model's temporal resolution (timestep) depends on the chosen granularity of driver datasets; however, observation operators allow for the assimilation of aggregated periods that extend beyond those driver timesteps. For example, CARDAMOM has assimilated long‐term mean leaf area index and annual net biosphere exchange values (Bloom et al. 2020; Quetin et al. 2020), despite operating at a monthly timestep in the cited examples. This flexibility allows CARDAMOM to capture ecological dynamics across various temporal scales. At daily resolution, CARDAMOM has differentiated ecosystem responses to grazing versus cutting practices (Myrgiotis et al. 2021). At monthly timesteps, it has captured ecosystem carbon dynamics, including net ecosystem exchange, when validated against withheld FLUXNET observations (Yang et al. 2022). At interannual timescales, it has accurately reproduced variability in net biosphere exchange and atmospheric CO_2_ growth rates (Bloom et al. 2020; Worden et al. 2024; Bilir et al. 2025). CARDAMOM has only been applied once at centennial scales using monthly timesteps (Quetin, Famiglietti, et al. 2023), and not without some difficulty (computational burden; lack of constraints prior to the 2000s). Lastly, a key downside is that CARDAMOM's parameters are time invariant, which can limit its ability to capture changes in ecosystems due to land use and land cover change (Milodowski et al. 2023) or the acclimation of plant traits to shifting climate conditions (Cui et al. 2020; Quetin, Anderegg, et al. 2023; Famiglietti et al. 2024).

CARDAMOM retrieves spatially localized DALEC parameters and initial conditions (e.g., wood pool value on first time step) based on the observations it assimilates. When applied at site scale (e.g., flux towers), separate CARDAMOM model‐data fusions are performed at each site to retrieve location‐specific parameters (e.g., Yang et al. 2022). Similarly, CARDAMOM can be applied across spatially contiguous domains, with model‐data fusion performed independently for each grid pixel, producing parameters and initial states specific to each location (e.g., Bloom et al. 2016). This approach allows parameters to vary across ecological gradients, capturing functional diversity as reflected in observations (e.g., Bloom et al. 2016; Smallman et al. 2017; Yin et al. 2020; Exbrayat et al. 2018; Smallman et al. 2021). Hence, unlike many large‐scale models, CARDAMOM avoids reliance on Plant Functional Types (hereafter PFTs) to define vegetation function. This is advantageous because PFTs capture only a fraction of plant trait variability and fail to account for significant intra‐PFT variability (Van Bodegom et al. 2012; Joswig et al. 2021; Konings et al. 2024).

#### 2.1.3 CARDAMOM Bayesian Model‐Data Fusion

CARDAMOM retrieves information on DALEC parameters and initial states based on assimilated observations, taking into account their quantity, type, and uncertainty (Smallman et al. 2017; Quetin et al. 2020; Williams 2022). A key feature of CARDAMOM is its explicit handling of uncertainty, treating parameters and outputs as random variables represented by probability distributions using a Bayesian approach. This incorporates both prior ecological knowledge and observational constraints to retrieve optimal parameter ensembles, following Bayes' Theorem:

$$p\left(x|O\right)\propto p\left(x\right)p\left(O|x\right)$$

Where *p*(*x*∣*O*) is the posterior probability distribution of a DALEC parameter vector *x*, given a set of observations *O*. The *p*(*x*) represents the prior knowledge of the parameter vector *x*, and *p*(*O*∣*x*) is the likelihood of the observations given the parameter ensemble.

The likelihood *p*(*O*∣*x*) is calculated using a cost function that compares observations (*O*) to their corresponding model state variables or fluxes generated from parameters *x*, scaled by an uncertainty term that accounts for both observational and model structural uncertainty (e.g., Bloom et al. 2016; Levine et al. 2025). In CARDAMOM, priors *p*(*x*) are divided into parameter priors *p*_par_(*x*) and ecological dynamic constraint (EDC) priors *p*_EDC_(*x*) (Figure 1), which integrate previously gathered information (e.g., past studies, literature, expert knowledge) into the optimization process. Parameter priors *p*_par_(*x*) are typically defined as uniform or log‐uniform distributions with bounds that exclude improbable or impossible values (e.g., negative turnover rates) but can be further constrained if parameter values are already known. EDC priors *p*_EDC_(*x*) embed ecological theory into model‐data fusion (Bloom and Williams 2015), reducing the parameter space by rejecting or penalizing parameter combinations that produce outcomes inconsistent with ecological principles. Commonly applied EDC priors include constraints on carbon allocation (e.g., balancing foliage and fine root allocation), turnover rates (e.g., slower turnover for soil organic matter compared to litter), and biomass dynamics (e.g., preventing exponential decay in foliage biomass). The number and type of EDC priors vary by model. EDC priors are removed when alternate structures reduce net dimensionality and are often introduced when new processes are added to DALEC.

Given the multidimensional parameter space in a DALEC model, solving the full Bayes equation analytically is impractical. Instead, numerical algorithms approximate the posterior distribution. CARDAMOM typically uses Markov Chain Monte Carlo (hereafter MCMC)‐based algorithms (e.g., Yang et al. 2022) but has also applied other samplers, such as simulated annealing (Myrgiotis et al. 2022). MCMC algorithms used in CARDAMOM include the Metropolis‐Hastings algorithm (Yang et al. 2022) and the Differential Evolution MCMC algorithm (Braak 2006; Worden et al. 2024). A key advantage of MCMC‐based parameter estimation approaches is that they quantify parametric uncertainty by generating a posterior distribution of parameters that reflects their likelihood given the data and model structure (Hill et al. 2012; Bloom et al. 2016).

### 2.2 History of the CARDAMOM Framework: Designed for Data Assimilation

CARDAMOM and its design philosophy originated from efforts to simplify the soil–plant‐atmosphere continuum model SPA (Williams et al. 1996), aligning it with the satellite observations and computational capabilities available at the time. This led to the development of an aggregate canopy model for gross primary productivity (Williams et al. 1997) and later the DALEC model (Williams et al. 2005). DALEC was designed to simulate carbon cycle dynamics in a manner optimized for data assimilation approaches (initially an ensemble Kalman filter) and to leverage the burgeoning availability of satellite observations (Williams et al. 2005). Building on DALEC, CARDAMOM was developed to explicitly infer parametric links between carbon model structures and observational data (Bloom and Williams 2015; Bloom et al. 2016; Quetin et al. 2020; Famiglietti et al. 2021; Smallman et al. 2021).

**FIGURE 2:**
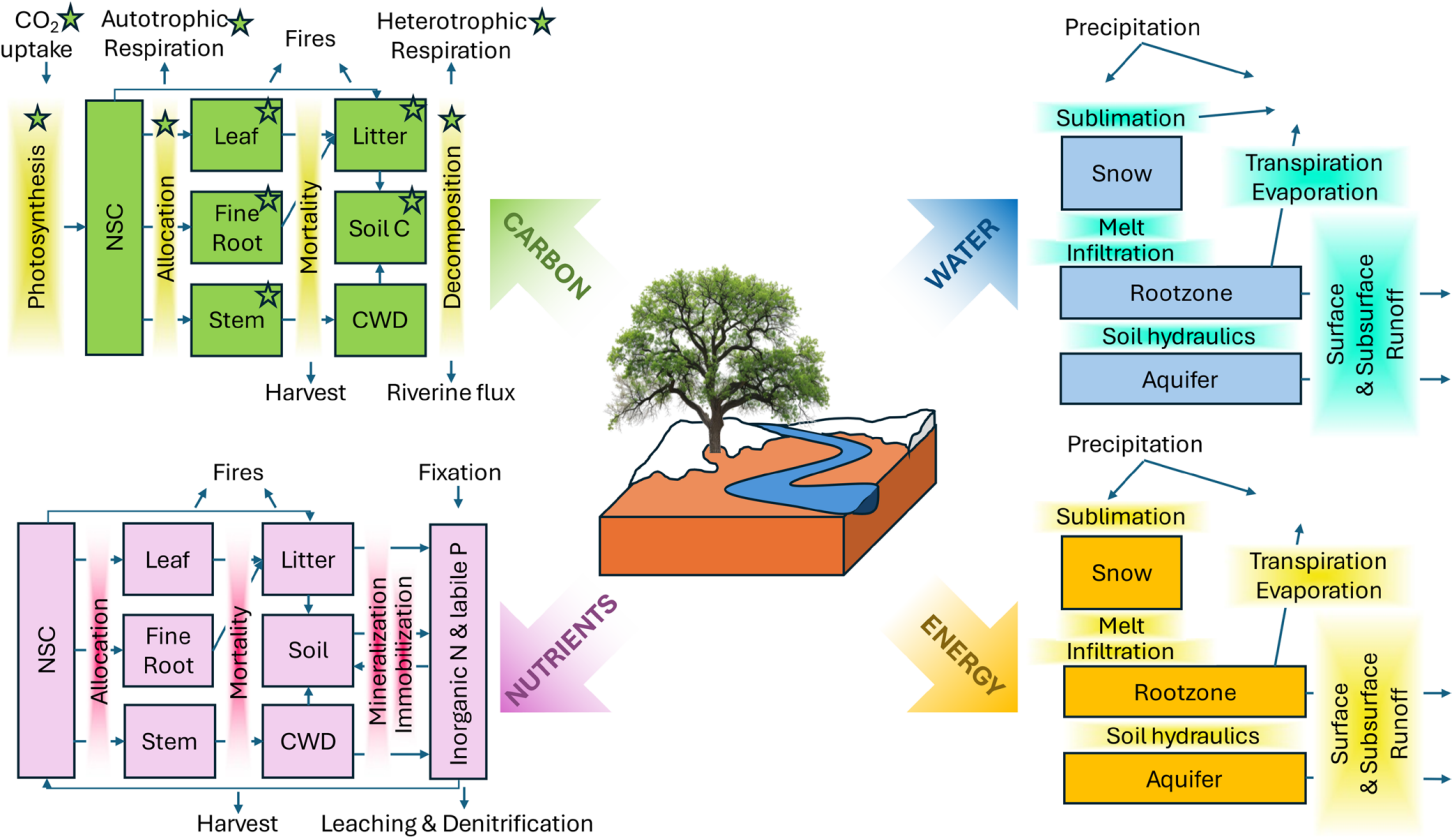
Schematic illustrating the range of DALEC ecosystem modules explored within the CARDAMOM community. The four panels depict the land surface carbon cycle (green, upper‐left), water cycle (blue, upper‐right), nutrient cycle (purple, lower‐left), and energy cycle (yellow, lower‐right). Within the respective carbon, water, nutrient, and energy cycles, boxes denote state variables, and arrows denote fluxes. No single DALEC variant currently integrates all four cycles at the maximum level of complexity; instead, different versions combine selected modules and implement them in distinct ways to address specific scientific questions. Green stars mark the DALEC's original scope, which covered only a subset of the carbon cycle (green panel, upper left).

CARDAMOM and DALEC are continuously developed across multiple institutions (currently the University of Edinburgh, Jet Propulsion Laboratory, Stanford University, Columbia University, University of California Davis, and University of California Santa Barbara) to address emerging scientific challenges and incorporate an expanding range of observational constraints. Over time, new DALEC versions have been developed, spanning a range of complexities and process representations—from carbon cycle models (e.g., Williams et al. 2005) to those incorporating hydrological, nutrient, and/or energy cycles (e.g., Wang et al. 2018; Levine et al. 2025; Bilir et al. 2025)—creating a suite of DALEC models (Figure 2). Concurrently, the number and type of observations assimilated by CARDAMOM have increased (Bloom et al. 2016; Famiglietti et al. 2023).

### 2.3 Applications to Extract Ecological Knowledge and Insight Into Model and Data Development

The CARDAMOM framework can be used for inferring ecosystem process information and for testing climate and management scenarios constrained by data. By assimilating localized observations, CARDAMOM can infer PFT‐agnostic and location‐specific ecosystem processes and traits at regional to global scales where direct measurement is infeasible. This capability has been leveraged in several ways, including for inferring carbon allocation, stocks, and turnovers (Bloom et al. 2016; Richardson et al. 2010; Rodríguez‐Veiga et al. 2020), changes in carbon allocation under drought (Au et al. 2023; Worden et al. 2024), management practices in grasslands (Myrgiotis et al. 2021), arable crop development and harvest (Revill et al. 2021), and temperature limitations on spring productivity in conifer forests (Stettz et al. 2022). Beyond capturing current ecosystem dynamics, CARDAMOM can also serve as a data‐constrained prognostic tool, simulating ecosystem responses to variables such as climate, CO_2_ levels, and disturbances. This is achieved by retrieving DALEC's parameters with present‐day observations and then running DALEC with modified forcing data or parameter distributions. This approach has been used to assess how declining fires in tropical ecosystems enhance the land carbon sink (Yin et al. 2020), explore lagged or legacy effects on carbon fluxes (Bloom et al. 2020; Worden et al. 2024), quantify carbon‐climate feedbacks in Alaskan wetlands (Ma et al. 2023), and partition the impacts of elevated CO_2_ versus climate change on forests in the Yucatán, Mexico (George‐Chacon et al. 2023), among others. These perturbation studies can be applied to both future and past projections. For example, one study quantified the impact of climate and CO_2_ fertilization over the past century on carbon sink distribution and found that respiration, a location‐specific optimized parameter, strongly influences whether a location acted as a source or sink (Quetin, Famiglietti, et al. 2023).

The design philosophy of CARDAMOM also makes it beneficial for model benchmarking. CARDAMOM's dual functionality allows it to be evaluated as a model (Yang et al. 2022; Quetin et al. 2020) while also serving as a coherent benchmark target when observations are unavailable, such as for permafrost soil residence times (Hugelius et al. 2024) or transit time distributions (Sierra et al. 2023). Alternative model structures can easily be compared to test hypotheses on process representation and its impact on modeled ecosystem sensitivity and predictive capacity (e.g., Famiglietti et al. 2021; Norton et al. 2023; Worden et al. 2024; Smallman et al. 2021). CARDAMOM outputs also provide valuable benchmark targets for other models by synthesizing multiple observation streams into an ecologically coherent representation of terrestrial ecosystems. This approach provides internally consistent estimates of carbon and water fluxes, stocks, and their associated uncertainties (Caen et al. 2022; Levine et al. 2023; Massoud et al. 2022). This capability is particularly important since different observational data streams may not be consistent with each other when compared naively. Examples of benchmarking include the use of CARDAMOM outputs in the international land model benchmarking (ILAMB) system (Collier et al. 2018; Friedlingstein et al. 2024), the validation of the Joint UK Land Environment Simulator (JULES) and Brazil's Integrated Land Surface Model (INLAND) land surface models across the Amazon (Slevin et al. 2017; Caen et al. 2022), and the validation of soil heterotrophic respiration in the Coupled Model Intercomparison Project Phase 6 (CMIP6) (Varney et al. 2022).

Finally, CARDAMOM can generate data‐constrained outputs of specific carbon fluxes that inform broader analyses and serve as a testbed for experiments aimed at improving model formulation and model‐data fusion. CARDAMOM outputs have been used to produce global satellite‐driven estimates of heterotrophic respiration (Konings et al. 2019) and provide prior ranges for atmospheric inversion estimates of net biosphere exchange (Liu et al. 2021). Examples of CARDAMOM as a testbed for models and model‐data fusion include assessing the impact of different analytical solvers, such as the Kalman filter versus genetic algorithms (Fox et al. 2009, a pre‐CARDAMOM DALEC‐based analysis), comparing model parameterization based on plant functional types to those derived from environmental filtering approaches (Famiglietti et al. 2023), and demonstrating how parametric error can dominate uncertainty in forecasts of carbon stock changes across Brazil (Smallman et al. 2021).

## 3 CARDAMOM's Spatially Explicit Parameters and Initial State Estimation Are Unique Among Data Assimilation Frameworks

**FIGURE 3:**
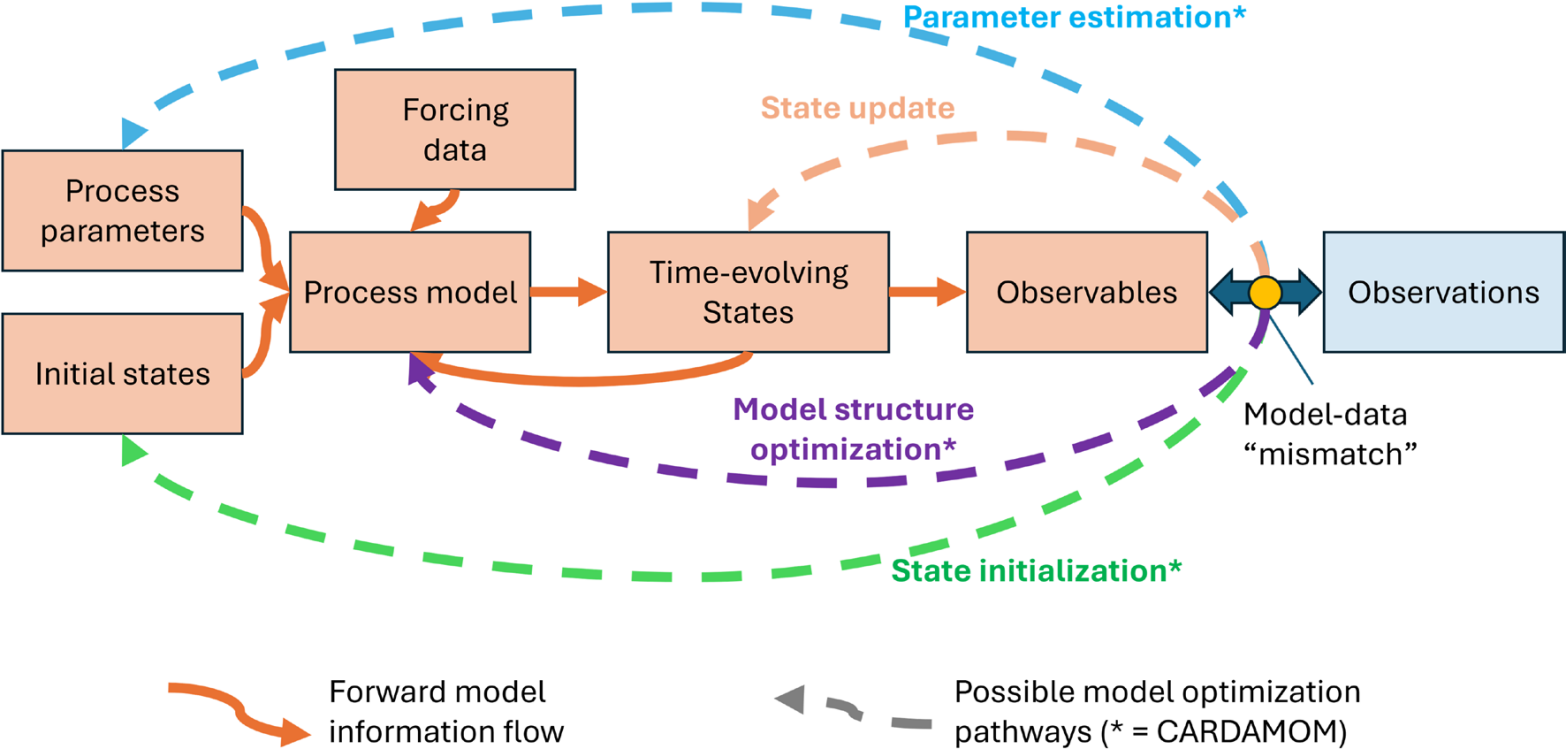
Process‐based model forward model information flow (orange solid arrows), linking parameters, initial conditions, and model forcing to observables. Model‐data fusion approaches propagate information from a model‐data “mismatch” assessment (e.g., a cost function and/or a probability evaluation) back to the model's configuration, through one or more model optimization “pathways” (dashed arrows). In contrast to other approaches, CARDAMOM optimizes time‐invariant process parameters and initial conditions on a local (point or gridscale) basis. See Table 1 for comparison of the CARDAMOM model‐data fusion approach relative to other state‐of‐the‐art approaches.

CARDAMOM is part of a growing set of frameworks that integrate process‐based models with ecosystem observations (Kaminski and Mathieu 2017). Across these frameworks, information generally flows in a similar pattern (Rayner et al. 2019; Figure 3). First, time‐invariant parameters and initial states drive a terrestrial biosphere model. Next, observation operators sample time‐varying states to generate “observables,” which are compared with actual ecosystem observations. Finally, any mismatch between these observables and observations is used to refine the process‐based model. Model‐data fusion typically follows one or more of four pathways: (1) sequential state update, (2) time‐invariant parameter estimation, (3) initial state estimation, and (4) model structure optimization (Figure 3).

The four pathways above offer various ways researchers can design frameworks (Table 1) to capture terrestrial biosphere processes accurately, incorporate observed ecosystem complexity, narrow parameter uncertainties, and produce data‐informed predictions (Luo et al. 2011; Williams et al. 2009; Li et al. 2024). State‐update approaches, such as the Community Land Model–Data Assimilation Research Testbed (CLM‐DART; Fox et al. 2018, 2022), sequentially adjust model states to match observations but do not refine model parameters, potentially violating mass conservation. In contrast, parameter retrieval approaches enhance ecological learning by estimating time‐invariant parameters that govern model processes. These retrievals can be spatially integrated to estimate parameters at the plant functional type level, as in the Carbon Cycle Data Assimilation System (CCDAS; Wu et al. 2020; Scholze et al. 2016; Norton et al. 2018), Organising Carbon and Hydrology In Dynamic Ecosystems Data Assimilation (ORCHIDEE‐DA; Kuppel et al. 2014; MacBean et al. 2022), and the Predictive Ecosystem Analyzer (PEcAn; LeBauer et al. 2013; Dokoohaki et al. 2021). They can also be spatially explicit, with parameters estimated locally, as in the Forest Biomass Assimilation and Regression (FöBAAR; Keenan et al. 2013), the Diagnostic Carbon Flux Model (DCFM; Xiao et al. 2014), and the Vegetation Photosynthesis and Respiration Model (VPRM; Mahadevan et al. 2008). The Climate Modeling Alliance (CliMA) Land, a more recent framework (Braghiere, Fisher, et al. 2021; Braghiere, Wang, et al. 2021; Wang et al. 2023), estimates both time‐invariant parameters and initial states while also updating states. However, each of these approaches either operates only over smaller spatial extents when data assimilation is performed, or if run at a global scale, solves for PFT‐wide parameters. Trade‐offs between model complexity, assimilation methodology, data availability, and computational constraints can make each framework better suited to specific scientific or practical applications.

**TABLE 1:** Comparison of the CARDAMOM model‐data fusion approach relative to other state‐of‐the‐art systems which integrate models and data.

Framework	Model‐data fusion algorithm or approach	Temporal and spatial resolution	State Update/initial state estimation	Process parameter estimation	Structure optimization	Prognostic with forcing data?	ESM integration
CLM‐DART	Bayesian	Diurnal, gridded	State Update	No	No	No	No
CCDAS (BETHY, SINDBAD, etc.…)	Bayesian, 4Dvar	Diurnal, gridded	No	Yes; PFT‐wide	No	Yes	Yes
ORCHIDEE‐DA	Gradient descent, genetic algorithm	Flexible	No	Yes; PFT‐wide	No	Yes	Yes
PEcAn	Bayesian	Flexible	State Update	Yes; PFT‐wide	No	Yes	No
CliMA‐Land	Ensemble Kalman Filter	Flexible	Both	Yes; PFT‐wide	No	Yes	Yes
DifferLand	Hybrid Model with gradient‐based optimizer	Flexible	Initial only	Yes	Yes	Yes	No
CARDAMOM	Bayesian (MCMC)	Flexible	Initial only	Yes	Yes^a^	Yes	No

^a^ Depending on how “structure” is defined, CARDAMOM can optimize model structure, such as retrieving different water stress or allocation functions using parametric equations (Levine et al. 2023; Worden et al. 2024).

CARDAMOM offers spatially explicit, PFT‐agnostic parameter and initial state optimization, carving out a distinct niche in model‐data fusion. While previous frameworks have explored spatially explicit parameter optimization in situ (e.g., Keenan et al. 2013; Xiao et al. 2014; Raj et al. 2018), CARDAMOM is, to our knowledge, the only framework operationalized on a gridded, global scale that retrieves both parameters and initial states. This spatially explicit approach enables independent estimation of process parameters like photosynthetic capacity, leaf mass per area, allocation fractions, and drought sensitivities alongside corresponding initial states like initial foliar biomass. By retrieving parameters across space rather than relying on predefined PFT‐level values (e.g., Bloom et al. 2016), CARDAMOM captures ecosystem functional gradients often overlooked by spatially integrated, PFT‐based approaches (e.g., Exbrayat et al. 2018). The combined state–parameter estimation paradigm, along with ecological and dynamic constraints (Bloom and Williams 2015; see Section 2.1.3), also provides a pathway to bypass multidecadal‐to‐millennial spin‐up times (e.g., Fang et al. 2015), which are both computationally expensive and depend on the assumption of initial equilibrium that may not be valid (Shi, M. et al., 2013).

CARDAMOM's ability to use data to constrain nonequilibrium initial conditions and spatially explicit parameters without relying on PFTs is unique relative to other data assimilation methods (Table 1). An exception is the recent DifferLand model (Fang and Gentine 2024). Built using DALEC and EDCs from CARDAMOM v2.3, DifferLand employs a hybrid physical–machine learning methodology that extends CARDAMOM's ability to optimize model structure by embedding neural networks within DALEC to replace existing empirical formulations. This includes, for example, replacing the soil water stress function used in gross primary productivity calculations with a neural network. The potential for hybrid and machine learning approaches to further extend CARDAMOM's capabilities is discussed in Section 5.4.

## 4 Model‐Data Fusion Challenges: Data Quality, Model, and Computation

### 4.1 Data Quality Propagates Into Inference

CARDAMOM relies on documented uncertainties, which are input alongside their corresponding observations (Section 2.1.3). However, it is often unclear how uncertainties in, for example, large‐scale dataset products should be scaled spatially and temporally when aggregated and used with CARDAMOM. Additionally, satellite and ecological network data are biased toward readily measurable features like canopy states, while biomass and dead organic matter—especially belowground components—often lack direct observational constraints. The uncertainty in these unobserved state variables is potentially substantial and can contribute significantly to forecast error within CARDAMOM (Smallman et al. 2021).

The interaction between these potentially large systematic errors in datasets, and inevitable structural errors in the model introduces a significant risk of biases being pushed into parameters that are less closely constrained by high‐density observation streams (Cameron et al. 2022). Attribution of changes in the carbon cycle to specific driving processes (and thus the parameterization of these processes) is further hindered by an incomplete view of the drivers (Exbrayat et al. 2018). For instance, forest degradation (Ryan et al. 2012) and herbivory (Metcalfe et al. 2014; Hempson et al. 2015) are major drivers of carbon turnover in tropical ecosystems; yet their extent and intensity are not as well mapped as deforestation (Hansen et al. 2010) or burned area (Andela et al. 2017).

### 4.2 Parameter Equifinality and Model Complexity

Equifinality is a challenge for all data assimilation approaches (Beven and Freer 2001). For a given set of observations, multiple combinations of model parameters and structures can produce simulations that fit ecosystem observations equally well (Famiglietti et al. 2021; Smallman et al. 2021). This limits model identifiability and becomes more pronounced as model complexity increases. While greater complexity (i.e., incorporating more processes) may enhance a model's ecological realism, without a corresponding increase in data constraints, parameter uncertainty rises, and forecast skill may decline (Famiglietti et al. 2021). Consequently, the guiding philosophy behind the development of DALEC models within CARDAMOM has been to adopt parsimonious, intermediate complexity models commensurate with available observations (Williams et al. 2005).

Developing and applying more complex models requires balancing ecological realism with the availability of observations needed to robustly characterize processes at relevant spatial and temporal scales. For example, dynamically partitioning heterotrophic respiration into aerobic and anaerobic components—important for understanding wetland carbon dynamics—requires appropriate observational constraints, such as methane emissions (Ma et al. 2023). Expanding the number and variety of observations assimilated by CARDAMOM is essential for improving model process representation and accuracy. However, the spatial and temporal resolution of these observations must align with model complexity. Coarser spatial scales and longer time steps (e.g., daily vs. monthly) may lack the necessary detail to constrain model processes effectively.

### 4.3 Temporal and Spatial Scale Mismatches Reduce Interpretability in Data Assimilation

The effectiveness of CARDAMOM is fundamentally shaped by the temporal and spatial scales of the assimilated data. Temporal aggregation affects the process signatures embedded in observations and influences how environmental drivers are characterized and propagated through CARDAMOM's land surface representation (Jenkins and Watts 1968; Grieve et al. 2016). For example, submonthly temporal resolution may be required to resolve drivers of interannual variability, such as the onset timing of the wet season (Camberlin and Okoola 2003), or short‐term management interventions (e.g., Myrgiotis et al. 2021).

Spatial resolution limitations present additional challenges. Coarse spatial scales average across diverse ecosystem types, degrading ecological information in retrieved parameters and potentially introducing bias into diagnostic analyses (Milodowski et al. 2023). In contrast, finer‐scale observations can resolve distinct processes driving ecosystem function, such as land management and disturbance (Fisher and Koven 2020). Environmental change hotspots (Alencar et al. 2015; Brinck et al. 2017) and managed, mixed‐use areas (Myrgiotis et al. 2022; Milodowski et al. 2023) are often characterized by heterogeneous, fragmented landscapes. The inability to resolve key management processes (e.g., arable harvest) has been identified as a factor contributing to discrepancies between bottom‐up terrestrial carbon balance estimates from land surface models and top‐down estimates from atmospheric inversions (White et al. 2019; Kondo et al. 2020), such as those often used to constrain CARDAMOM.

The spatial resolution of a CARDAMOM analysis is limited by its coarsest dataset. CARDAMOM assimilates data types with widely varying spatial resolutions—for example, net biosphere exchange derived from atmospheric inversions typically has a resolution > 100 km, while leaf area index or biomass data may have resolutions several orders of magnitude finer (10–500 m) and must be aggregated to use alongside net biosphere exchange. As a result, there is a trade‐off between the spatial resolution needed to address a given research question and the dimensionality of available observational constraints, linking back to the equifinality challenge outlined in Section 4.2. An outstanding challenge is to resolve the ecological structure of heterogeneous landscapes while maximizing the use of available observation constraints from a multiscale satellite‐based observation network. Besides acquiring higher‐resolution observations, addressing this limitation could involve refining existing aggregation methods (e.g., stratifying assimilated data based on land use distributions; Milodowski et al. 2023) or developing new approaches, such as hierarchical models or novel tiling techniques (e.g., Hill et al. 2011), that enable cross‐scale integration of observations in CARDAMOM's likelihood calculation.

### 4.4 Computational Cost

CARDAMOM currently uses MCMC‐based algorithms, which can require significant computational effort. Each CARDAMOM location or pixel requires 10^5^–10^9^ model simulations to reach convergence. This computational cost is mitigated by DALEC's intermediate complexity, for which individual simulations are computationally inexpensive (Bloom and Williams 2015; Bloom et al. 2016; Famiglietti et al. 2021). As a result, single‐site analyses can be performed on a standard workstation. Scaling up to large spatial domains, however, requires high‐performance computing (HPC) or cloud‐based architectures to run CARDAMOM at each point concurrently, introducing tradeoffs in the spatial and temporal resolution of regional to global analyses.

## 5 Community Recommendations

### 5.1 Incorporating New Observations and Processes

**FIGURE 4:**
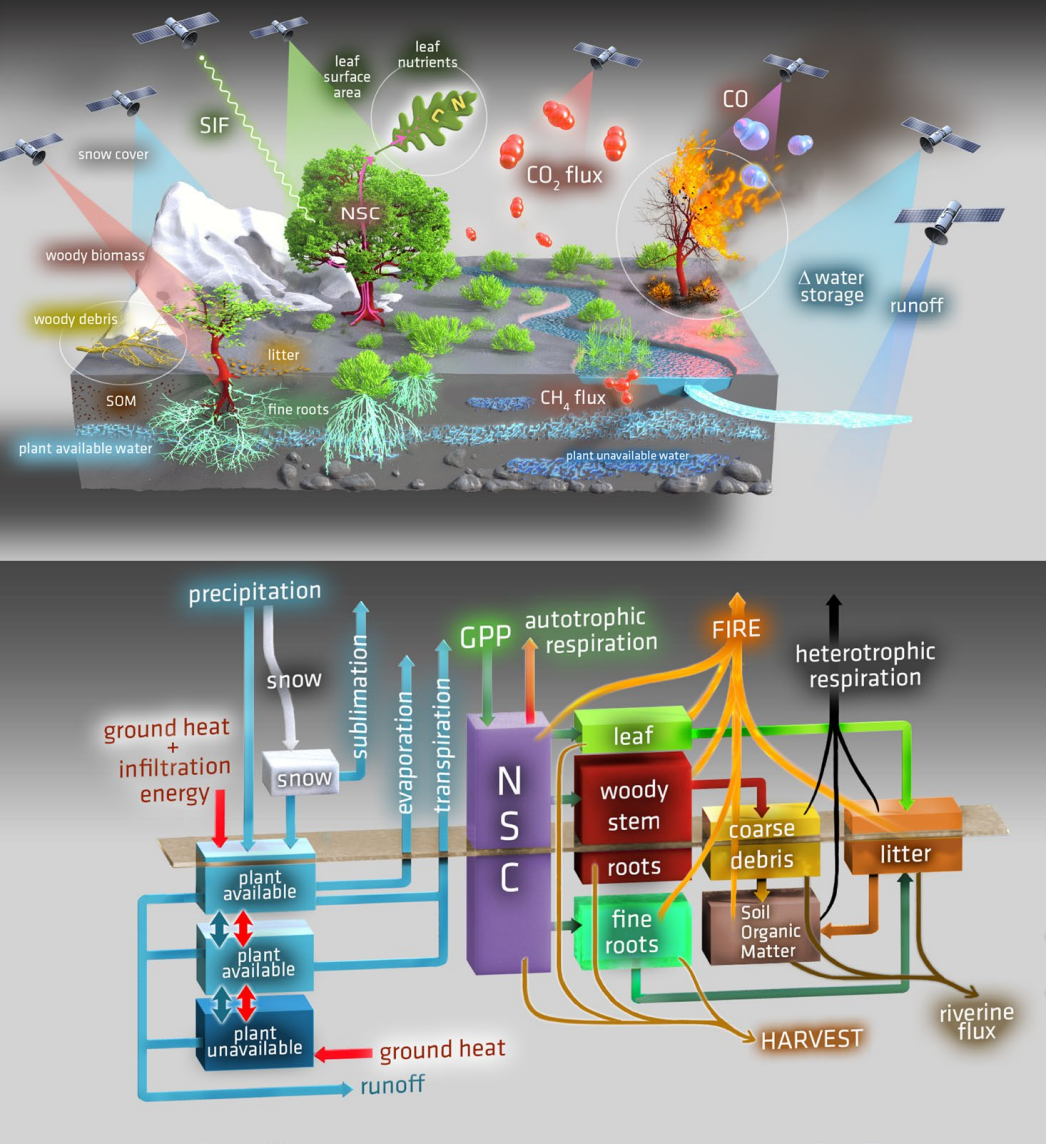
Above figure shows current and future satellite‐based observations used by CARDAMOM to constrain DALEC. The figure below presents a schematic representation of DALEC corresponding to the ecosystem shown in the figure above. Illustration by Victor O. Leshyk.

CARDAMOM's flexibility in assimilating different observation streams and its adaptable model structure make it well‐suited to leveraging the expanding array of ecosystem observations from space (Figure 4). Globally, it can already assimilate datasets on net biosphere exchange (e.g., NASA Carbon Monitoring System Flux [CMS‐Flux]; Liu et al. 2021), equivalent water thickness (e.g., Gravity Recovery and Climate Experiment [GRACE]; Watkins et al. 2015), fire carbon emissions (e.g., Measurements of Pollution in the Troposphere [MOPITT]; Jiang et al. 2017; Global Fire Emissions Database [GFED]; Giglio et al. 2013), soil organic carbon stocks (e.g., SoilGrids v2; Poggio et al. 2021; Harmonized World Soil Database [HWSD]; Hiederer et al. 2011), leaf area index (e.g., Moderate Resolution Imaging Spectroradiometer [MODIS]; Myneni et al. 2015), methane emissions (e.g., Wetland and Wetland CH_4_ Emissions Model Intercomparison Project [WetCHARTs]; Ma et al. 2021), aboveground biomass (e.g., Xu et al. 2021; ESA Climate Change Initiative Biomass [ESA‐CCI Biomass]; Santoro and Cartus 2024), and solar‐induced fluorescence (e.g., Joiner et al. 2018; Sun et al. 2018).

A growing number of recent and forthcoming observational platforms offer opportunities to provide additional constraints for CARDAMOM (Table 2). These include the NASA–ISRO Synthetic Aperture Radar (NISAR; Kellogg et al. 2020), BIOMASS (Quegan et al. 2019), the Global Ecosystem Dynamics Investigation (GEDI; Dubayah et al. 2023), and the Radar Observing System for Europe L‐band (ROSE‐L; Davidson and Furnell 2021) missions. Their emphasis on calibration, error characterization, and repeated aboveground biomass estimates will enhance CARDAMOM's ability to capture biomass growth and disturbance dynamics. Additionally, the Copernicus Anthropogenic Carbon Dioxide Monitoring mission (CO2M; Noël et al. 2024) will provide new estimates of column CO_2_ and methane, enabling surface net carbon flux derivations that can be used to constrain CARDAMOM. CO2M, along with the FLuorescence EXplorer (FLEX; Moreno 2021), will also contribute new solar‐induced fluorescence measurements. The recent Surface Water and Ocean Topography (SWOT) mission (Fu et al. 2024) is expected to deliver global river discharge estimates (Durand et al. 2023), which, on a monthly scale, can be converted to runoff maps (Pan and Wood 2013; David et al. 2019; Collins et al. 2024). These runoff maps could help constrain spatial and temporal runoff variability in CARDAMOM, which has historically been difficult to validate.

**TABLE 2:** Possible new datasets for assimilation into CARDAMOM include existing, previously unassimilated datasets as well as those from future missions.

Mission	Measurement	Availability	Source
GEDI	Forest biomass	Available	Dubayah et al. (2023)
SWOT	Surface water extent and elevation	Available	Fu et al. (2024)
AVIRIS	Hyperspectral‐derived plant traits	Available	Townsend et al. (2023)
EMIT	Hyperspectral‐derived plant traits	Available	Green et al. (2020)
NISAR	Forest biomass	Recently launched	Kellogg et al. (2020)
BIOMASS	Forest biomass	Recently launched	Quegan et al. (2019)
FLEX	Solar‐induced fluorescence and plant stress	2026	Moreno (2021)
CO2M	Total column CO_2_ and CH_4_. Solar‐induced fluorescence	2027	Noël et al. (2024)
ROSE‐L	Forest biomass	2027	Davidson and Furnell (2021)
CHIME	Hyperspectral‐derived plant traits, and soil surface carbon and texture	2028	Rast et al. (2021)
SBG	Hyperspectral‐derived plant traits	2032	Schimel and Poulter (2022)

Hyperspectral satellite data offer new opportunities to constrain leaf properties. These constraints can improve modeling of phenology, gross primary productivity, and autotrophic respiration. Current observations of foliar nitrogen content from the Airborne Visible/Infrared Imaging Spectrometer (AVIRIS; Townsend et al. 2023), the Earth Surface Mineral Dust Source Investigation (EMIT; Green et al. 2020), and the Plankton, Aerosol, Cloud, ocean Ecosystem mission (PACE) will be complemented by the upcoming Copernicus Hyperspectral Imaging Mission for the Environment (CHIME; Rast et al. 2021) and Surface Biology and Geology (SBG; Schimel and Poulter 2022) missions (Table 2). When combined with already assimilated observations, these measurements can refine theoretical models linking ecosystem carbon and nitrogen cycles (e.g., Thomas and Williams 2014; Flack‐Prain et al. 2019; Thomas et al. 2019). Hyperspectral data also enhance representations of leaf lifespan via leaf mass per area and photosynthetic capacity via leaf chlorophyll content. Additionally, hyperspectral signals correlate with mycorrhizal fungi (Sousa et al. 2021), which play a key role in plant nutrient acquisition and responses to CO_2_ fertilization (Braghiere, Fisher, et al. 2021; Braghiere, Wang, et al. 2021; Braghiere et al. 2022). These fungi form symbiotic relationships with most plants, enhancing their nutrient acquisition—particularly nitrogen and phosphorus—which is essential for plant growth (Shi et al. 2023). These data present an opportunity to incorporate fungal dynamics into CARDAMOM, advancing our understanding of their influence on nutrient cycling and plant productivity.

Beyond exploiting the growing catalogue of remote sensing observations, CARDAMOM excels at extracting ecological insights from a broad range of observational networks and experimental platforms. For example, forest chronosequence data can constrain biomass accumulation rates in DALEC (Smallman et al. 2017; George‐Chacon et al. 2023). Forest inventory data—such as from the Red Amazónica de Inventarios Forestales (RAINFOR) network (Malhi et al. 2002)—provide robust estimates of forest states. CARDAMOM can assimilate these data at the site level to refine allocation and turnover rates (Ge et al. 2022). Additionally, global plant trait databases, such as the Fine‐Root Ecology Database (FRED; Iversen et al. 2017) and the TRY Plant Trait Database (TRY; Kattge et al. 2020), provide valuable constraints for prior parameter estimates and refined ecological dynamic constraints. Manipulation experiments involving drought or CO_2_ adjustments offer further opportunities to refine vegetation models (Medlyn et al. 2015). By calibrating DALEC with CARDAMOM under both control and intervention scenarios, hypotheses about functional adaptation under stress can be tested, with predictive uncertainties propagated into ecosystem forecasts under climate and CO_2_ change.

### 5.2 Engagement With Wider Remote Sensing, Field, and Land Surface Modeling Communities

CARDAMOM does not exist in isolation, and its community must engage strongly with other research communities to maximize the possibility for ecological learning and supporting the creation of actionable information that can support decision‐makers. Three directly relevant communities are remote sensing scientists, field scientists, and land surface modellers.

As new satellite missions and products emerge, fostering closer engagement with the remote sensing community remains a priority. CARDAMOM requires robust uncertainty estimates to guide assimilation effectively. Developing these estimates collaboratively with teams producing remote sensing products is essential (Labrière et al. 2023). CARDAMOM users would benefit significantly from a better understanding of how uncertainties scale with spatial aggregation and vary across time. Furthermore, observation operators that link DALEC state variables to observables (Reyes‐Muñoz et al. 2024) may vary spatially and temporally due to factors such as ecological heterogeneity and human management, requiring input from remote sensing product creators. Additionally, the CARDAMOM community has substantial opportunities to shape future satellite missions by conducting data assimilation experiments with existing (Smallman et al. 2017, 2021) and synthetic datasets (Holtzman et al. 2023) to refine observation requirements and quantify the potential value of observations.

Close collaboration with field communities also offers an opportunity for improving process understanding from site to ecosystem scale. Efforts are increasing to establish networks of field inventory sites that provide consistent, repeated ecological observations (e.g., Malhi et al. 2002, 2021; Lopez‐Gonzalez et al. 2011; The SEOSAW partnership 2021). These networks could benefit from systematic, data‐informed analyses to generate and evaluate hypotheses relevant to the ecosystems they monitor (e.g., Medlyn et al. 2016).

The land surface modeling community supports national decision‐makers (e.g., national carbon budgets) and international climate negotiations (e.g., IPCC reports) through large multimodel ensembles, such as the Trends in Global Net Land‐Atmosphere Carbon Exchange project (TRENDY; Sitch et al. 2024). However, these models, due to their size and complexity, struggle to ingest observations for parameter updates and improved predictions. CARDAMOM offers a useful alternative by providing observationally informed insights into ecosystem states, dynamics, and traits, enabling more rigorous, systematic model evaluation, and identifying pathways for improvement (e.g., Caen et al. 2022; Williams et al. 2024). CARDAMOM already provides data‐constrained carbon fluxes to TRENDY (e.g., Friedlingstein et al. 2023, 2024) and the Regional Carbon Cycle Assessment and Processes Phase 2 project (RECCAP2; Jones et al. 2023; Hugelius et al. 2024). Strengthening these connections will potentially expand CARDAMOM's usage and foster broader engagement between communities.

### 5.3 Supporting Localized Ecosystem Monitoring, Management, and Decision‐Making

CARDAMOM readily adapts to distinct terrestrial ecosystems, simulating responses to climate, management, and disturbance scenarios while propagating uncertainties for robust confidence estimates. It has already successfully modeled land management impacts in forestry (Smallman et al. 2017), arable ecosystems (Revill et al. 2021), and pastures (Myrgiotis et al. 2021, 2022). Additionally, its ability to assess shifting fire regimes, drought impacts, and landuse changes is highly relevant to land management and policy. When applied at appropriate spatial scales, these capabilities offer significant opportunities to support decision‐makers and land managers with actionable insights for sustainable ecosystem management, such as simulating forest response to deforestation or disturbance (Smallman et al. 2022; Levy et al. 2022). The DALEC model structure also aligns with IPCC guidelines for United Nations Framework Convention on Climate Change (UNFCCC) national emissions reporting (IPCC 2006). By providing localized, observation‐informed, and uncertainty‐bounded carbon budgets, it offers an opportunity to enhance Tier 2 and Tier 3 land carbon balance reporting (Williams et al. 2024).

While CARDAMOM and DALEC show strong potential for supporting management, challenges remain. First, integrating CARDAMOM into operational frameworks requires collaboration with stakeholders (Melo et al. 2023). Second, capturing fine‐scale land management patterns is difficult in large‐scale studies relying on coarse spatial resolution (> 1 km) observations (Milodowski et al. 2023). Third, robust frameworks for simulating landuse changes, especially in dynamic hotspots or over long periods, may require the development of temporal adjustments to model parameters to account for processes like biodiversity shifts (George‐Chacon et al. 2023), which have not yet been common among CARDAMOM usage.

### 5.4 Augmenting CARDAMOM With Machine Learning

**FIGURE 5:**
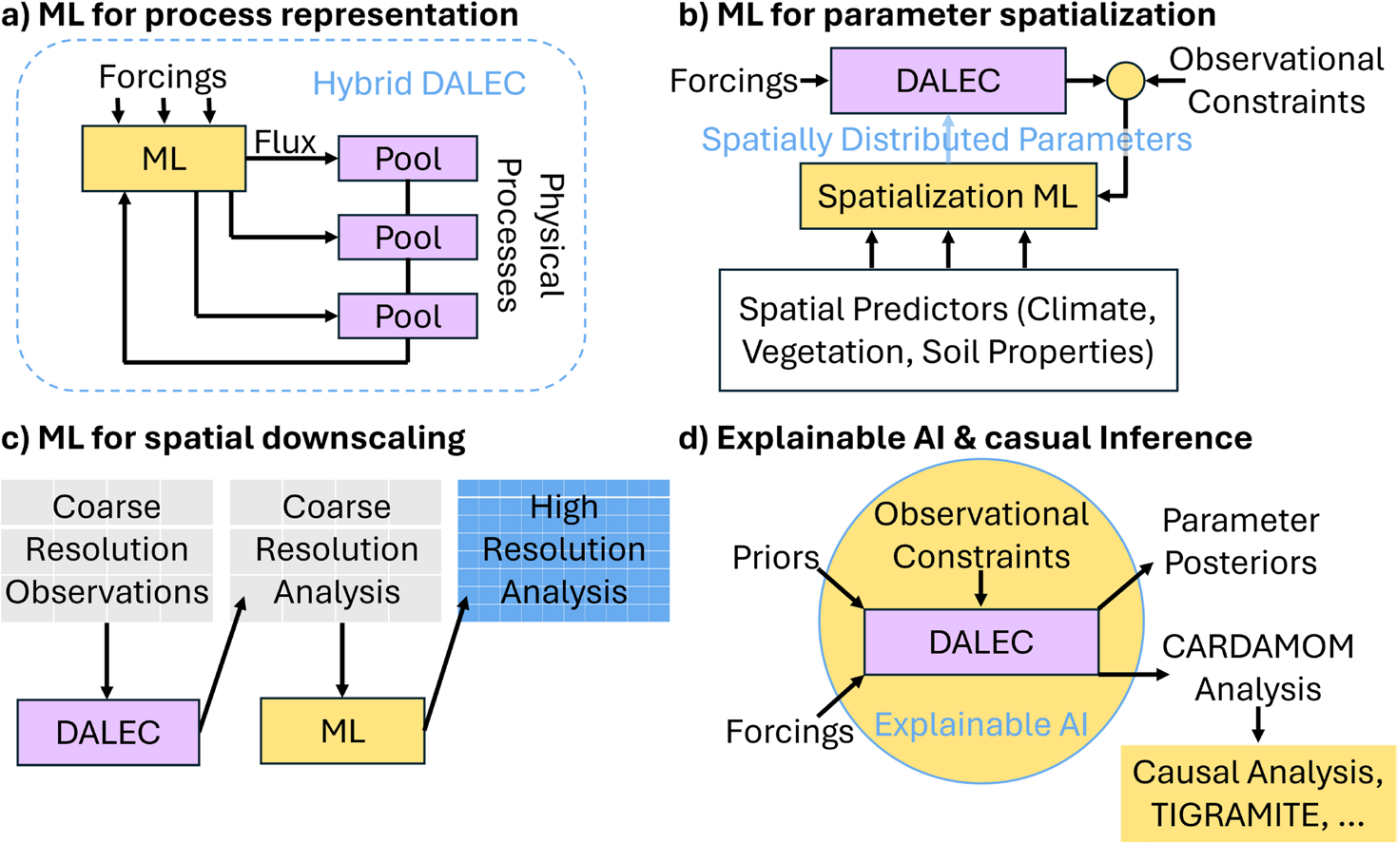
Schematic showing different ways that machine learning (ML) can be incorporated into the CARDAMOM framework. (a) A hybrid model using machine learning to represent unknown functional relationships within a DALEC model; (b) A spatialization machine learning model used to simulate the relationships between observable climate, vegetation, and soil properties and the latent ecological variables in CARDAMOM; (c) machine learning used to produce high‐resolution analyses of key ecological variables based on coarse‐resolution CARDAMOM output; (d) Using interpretable AI and causal analysis tools in conjunction with CARDAMOM to understand the links between different ecological processes.

The CARDAMOM framework is well‐suited for integrating machine learning and deep learning approaches, while minimizing their drawbacks. Unlike purely data‐driven techniques, DALEC models prognostically evolve carbon and water pools, enforcing mass conservation and allowing ecological memory effects to emerge in state‐dependent processes (Bloom et al. 2020; Reichstein et al. 2019). CARDAMOM's ecological dynamic constraints and prior distributions eliminate many physically and ecologically infeasible parameter combinations, effectively reducing the dimensionality of the parameter space. This regularization allows the model to perform well even with sparse observations (Bloom and Williams 2015; Famiglietti et al. 2021). While traditional machine learning models often require thousands of training samples for robust performance, CARDAMOM analyses can achieve strong results with as few as 10–100 observations (Bloom et al. 2016; Shen et al. 2023). Building on these strengths, current and emerging machine learning strategies can complement and enhance CARDAMOM, including, for example, new architectures using attention mechanisms (Vaswani et al. 2017), transfer learning (Immorlano et al. 2025), and hybrid model architectures (Fang and Gentine 2024). Below we describe four strategies through which machine learning techniques are currently or planned by the CARDAMOM community to be combined with CARDAMOM to enhance its utility (Figure 5).

First, machine learning can be used to fit unknown functional relationships within terrestrial biosphere models by replacing a prescribed model component with an embedded machine learning submodule, resulting in a hybrid model (Figure 5a). This approach is particularly effective for biophysical processes that are fast and have ample observational data (Reichstein et al. 2019; Braghiere et al. 2020; Massoud et al. 2023). The machine learning parameters (e.g., weights and biases in a neural network) can be calibrated alongside the physical parameters in a DALEC model, allowing for simultaneous quantification of structural and parametric uncertainties. For example, neural networks have been used to parameterize site‐specific empirical water stress functions in a DALEC model, accounting for photosynthesis downregulation due to soil moisture limitation or high atmospheric demand (Fang and Gentine 2024). This is advantageous as uncertainties in the functional form of water stress contribute significantly to global carbon budget uncertainties (Trugman et al. 2018).

Second, DALEC can be coupled with a spatialization neural network to predict model parameters from spatial variables like climate and topography (Figure 5b; Reichstein et al. 2022; Bao et al. 2023; ElGhawi et al. 2023), though other approaches exist (Famiglietti et al. 2023). This coupled ML model (Fang et al. 2024) treats DALEC as a “physical layer” that learns parameters as a function of ecological and climate properties at each location. Parameter updates occur via gradients derived from the mismatch between DALEC simulations and observational data constraints, with tools like JAX, Julia, or PyTorch computing these gradients (Shen et al. 2023). This method offers two key benefits: (1) It preserves spatial covariations in model parameters imposed by model structures, forcing variables, and observational constraints, reducing overfitting compared to pixel‐wise fitting with limited data; and (2) it incorporates the parameter sensitivity of the process‐based model into the neural network through backpropagation, refining parameters while preventing infeasible combinations that could lead to instability or nonphysical outputs (Fang et al. 2024). Applying this approach to a light‐use efficiency photosynthesis model has improved out‐of‐sample predictions, suggesting broader applicability in terrestrial biosphere modeling (Bao et al. 2023, 2024), including CARDAMOM applications.

Third, machine learning could downscale CARDAMOM outputs, generating higher‐resolution ecological maps than the spatial resolution of CARDAMOM assimilations (Figure 5c). These fine‐scale maps can support land management decisions and ecological service assessments (Yu et al. 2022). Currently, CARDAMOM's spatial resolution is constrained by computational capacity or the resolution of the coarsest dataset (see Section 4.3); machine learning offers a way to overcome these limitations by producing fine‐grained estimates constrained by coarse‐grained ones (Reichstein et al. 2019).

Fourth, machine learning techniques provide new ways to causally interpret (Bareinboim and Pearl 2016; Bareinboim et al. 2022; Massmann et al. 2021) CARDAMOM behavior (Figure 5d). Applying causal analysis to spatial CARDAMOM analyses can reveal biogeographic controls on the carbon cycle, leveraging CARDAMOM's ability to synthesize diverse observations into systemically consistent ecosystem representations. This integration helps capture potentially confounding mechanistic pathways that individual observation streams may not fully resolve. Spatial CARDAMOM analyses can be combined with existing causal inference frameworks, such as Time‐series Graph‐based Measures of Information Transfer (TIGRAMITE; Runge 2018; Runge et al. 2023), to explore biogeographic variations. Applying causal analysis to a CARDAMOM representation of southern African woodlands demonstrated how interactions between climate, fire, and ecosystem traits drive spatial differences in carbon stocks, residence times, and fluxes (Williams et al. 2024).

As machine learning is incorporated into CARDAMOM, it would benefit from being benchmarked against Bayesian data assimilation‐based versions in terms of predictive performance, parameter inference (e.g., spatial coherence and accuracy in retrieved ecosystem parameters across grids), computational efficiency (e.g., runtime and resource usage on high‐performance computing clusters), uncertainty quantification capabilities (e.g., ensemble spread in model outputs and posterior distributions of parameters), and the ability to capture extreme events (e.g., droughts, floods, and heatwaves). Both versions would further benefit from broader benchmarks against existing process‐based models (e.g., Quetin et al. 2020) as well as contemporary and emerging machine learning approaches (Dueben et al. 2022; Ullrich et al. 2025). Specific machine learning‐based Earth system models for these comparisons could include random forest or neural network ensembles for estimating land surface fluxes (e.g., FLUXCOM; Jung et al. 2020), transformer‐based architectures (e.g., Nguyen et al. 2023; Szwarcman et al. 2024), physics‐informed neural networks (e.g., Wang et al. 2022), and foundation models pretrained on extensive climate datasets (e.g., Bodnar et al. 2025). While a standardized benchmark does not yet exist, Dueben et al. (2022) provide examples for machine learning‐inspired evaluations, and Ullrich et al. (2025) mention potential standards for such benchmarks while encouraging leveraging existing process‐based intercomparisons. Complementary comparisons against intercomparison frameworks could include the use of the International Land Model Benchmarking (ILAMB; Collier et al. 2018; already incorporated with the Bayesian version of CARDAMOM) and the Earth System Model Evaluation Tool (ESMValTool; Lauer et al. 2025).

## 6 Conclusions

The potential for integrating new datasets and machine learning approaches into CARDAMOM presents opportunities to extract ecological insights across both site and global scales, as well as enhance model process representation. However, challenges such as data quality, computational costs, parameter equifinality, and model complexity must be understood to fully realize this potential. Placing CARDAMOM within the broader context of data assimilation frameworks reveals its unique strengths in spatially explicit, PFT‐agnostic optimization of initial states and parameters on a global scale. To capitalize on these strengths, we recommend further collaboration with remote sensing, field, and land surface modeling communities to refine uncertainty estimates and deepen ecosystem understanding. These efforts can also deliver observation‐informed, localized insights that support sustainable management and policy decisions. CARDAMOM's continued evolution will help bridge the gap between observations and process‐based models, advancing our understanding of terrestrial ecosystem dynamics under a changing biosphere.

## Author Contributions

**Matthew A. Worden:** conceptualization, project administration, visualization, writing – original draft, writing – review and editing. **T. Eren Bilir:** conceptualization, project administration, visualization, writing – original draft, writing – review and editing. **A. Anthony Bloom:** conceptualization, project administration, visualization, writing – original draft, writing – review and editing. **Jianing Fang:** conceptualization, project administration, visualization, writing – original draft, writing – review and editing. **Lily P. Klinek:** conceptualization, project administration, writing – original draft. **Alexandra G. Konings:** conceptualization, supervision, visualization, writing – original draft, writing – review and editing. **Paul A. Levine:** conceptualization, project administration, visualization, writing – original draft, writing – review and editing. **David T. Milodowski:** conceptualization, writing – original draft, writing – review and editing. **Gregory R. Quetin:** conceptualization, visualization, writing – original draft, writing – review and editing. **T. Luke Smallman:** conceptualization, visualization, writing – original draft, writing – review and editing. **Yinon M. Bar‐On:** conceptualization, writing – original draft. **Renato K. Braghiere:** conceptualization, writing – original draft, writing – review and editing. **Cédric H. David:** writing – review and editing. **Nina A. Fischer:** conceptualization, writing – original draft. **Pierre Gentine:** writing – review and editing. **Tim J. Green:** conceptualization, writing – original draft. **Ayanna Jones:** writing – original draft. **Junjie Liu:** writing – review and editing. **Marcos Longo:** writing – review and editing. **Shuang Ma:** writing – review and editing. **Troy S. Magney:** conceptualization, writing – original draft, writing – review and editing. **Elias C. Massoud:** conceptualization, writing – original draft, writing – review and editing. **Vasileios Myrgiotis:** writing – review and editing. **Alexander J. Norton:** conceptualization, writing – original draft. **Nick Parazoo:** conceptualization, writing – original draft. **Elahe Tajfar:** conceptualization, writing – original draft. **Anna T. Trugman:** conceptualization, writing – original draft, writing – review and editing. **Mathew Williams:** conceptualization, writing – original draft, writing – review and editing. **Sarah Worden:** conceptualization, writing – original draft, writing – review and editing. **Wenli Zhao:** conceptualization, writing – original draft. **Songyan Zhu:** conceptualization, writing – original draft, writing – review and editing.

## Conflicts of Interest

The authors declare no conflicts of interest.

## Data Availability

Data sharing not applicable to this article as no datasets were generated or analyzed during the current study.
